# Electricity—Its Uses and Abuses

**Published:** 1875-09

**Authors:** E. H. Gibbs


### ELECTRICITY—ITS USES AND ABUSES.

BY E. H. GIBBS, M. D.

The change that has taken place in the views of educated physicians in regard to the employment of electricity as a hygienic and curative agent, is very remarkable. I recently called on my preceptor and friend, who is one of the most eminent surgeons in this country, and whose books are standard works with the profession. I had not seen him for years. Knowing that he regarded with contempt anything that deviated from what is called "regular practice," I hesitatingly informed him, that I believed in electricity, and was employing it in at least four cases out of five in my practice, with the most satisfactory results. I was as much surprised as gratified when in reply he expressed the opinion that electricity was the best remedial agent known, and applicable to a greater variety of cases than any other. Pointing to an electrical instrument, he said, "You see the evidence of my faith in electricity, but I find but little time to use it, and in future I shall send to you such of my patients as require that treatment." He said that nearly all physicians recognized it as too important and powerful a remedy, to be monopolized by charlatans and ignorant pretenders who do not understand how to employ it judiciously, and therefore do great injury

In the incipient stages of many diseases of less or greater fatality, electricity shows its most wonderful power.

We every day see it verifying the truth of that old proverb "an ounce of prevention is worth a pound of cure." Any one can point out diseases that have become incurable through neglect, probably most of which could have been arrested in a few hours or days by prompt attention. I do not claim that electricity will arrest the march of all diseases, but it will cure a great many. All nervous affections, and particularly those of the spine, chest and limbs, yield more readily to electricity when properly applied, than to any other treatment. The wonder is; that intelligent people will be conscious of the approach of some disease affecting a vital part, as the brain, heart, lungs, kidneys or liver, and neglect to avail themselves of those remedies that science has placed within their reach.

As to the abuses of electricity, but few words are necessary. He who prescribes "hap-hazard", administering remedies without a specific object, only for the sake of seeming to do something, is a quack, and unworthy of confidence. Nearly all remedies debilitate and do harm and the good to be expected must be sufficient to outweigh that Electricity is a powerful remedy, and should be employed with as much care and skill as any other. Some of the cases in which we may expect decided benefit from the proper administration of electricity, are; Ague, Aneurism, Angina-pectoris, Constipation, Chorea, Debility, Headache, Hysteria, Neuralgia, Palsy and all Nervous affections, Rheumatism, Skin Diseases, Spinal Irritation, Swellings and Tumors—all of which have a general character, and with one or two exceptions are affections of parts deeply seated, or consist of deposits and enlargements, as in Glands and Joints.

---

Dr. Gibbs' office is at No. 137 Eighth street, between Broadway and 4th Avenue. Office hours from 10 A. M. to 4 P. M.

